# The Treatment of Gout by Alkalies

**Published:** 1898-06-18

**Authors:** 


### THE TREATMENT OF GOUT BY ALKALIES.

As the result of a careful series of experiments in regard to the action of alkalies in gout, Dr. Arthur P. Luff comes to the conclusion (*a*) that the ordinary alkalies, the lithium salts, piperazine, and lysidine, do not exercise any special solvent effect on sodium biurate, and their administration with the object of removing uratic deposits in the joints and tissues appears to be useless, and (*b*) that sodium salicylate does not exercise any special solvent effect on sodium biurate; its administration with the object of removing uratic deposits in the joints and tissues appears to be useless, and, moreover, it is apparently contra-indicated in gout on account of its leading to an increased formation of uric acid in the kidneys. It must, however, be noted that Dr. Luff's investigations were made by means of laboratory experiments, and although these appear, for the time being, to knock on the head the theory which has in many cases been the excuse for the administration of these remedies, they by no means touch the fact that many patients suffering from various manifestations of gout are better both in health and temper while taking alkalies. How these act is another matter. Dr. Luff has shown that they do not act in the way that they have been commonly supposed to do, but there is nothing in his paper to show that they have no beneficial influence. This must be decided by clinical observation.

